# Functionalized Erythrocyte Membrane-Coated Nanoparticles for the Treatment of *Klebsiella pneumoniae*-Induced Sepsis

**DOI:** 10.3389/fmicb.2022.901979

**Published:** 2022-06-16

**Authors:** Jun Liu, Hui Ding, Mingjie Zhao, Fan Tu, Tian He, Lizhu Zhang, Yanfei Jing, Xiaohong Rui, Shiliang Zhang

**Affiliations:** ^1^Department of Laboratory Medicine, Wuxi Fifth People’s Hospital Affiliated to Nantong University, Wuxi, China; ^2^Department of General Medicine, Wuxi Fifth People’s Hospital Affiliated to Nantong University, Wuxi, China; ^3^Nanxin Pharm, Nanjing, China; ^4^Department of Function, Wuxi Fifth People’s Hospital Affiliated to Nantong University, Wuxi, China

**Keywords:** sepsis, *Klebsiella pneumoniae*, γ3 peptide, targeted therapy, red blood cell membrane

## Abstract

Sepsis is a systemic inflammatory response syndrome caused by infection, with high incidence and mortality. Therefore, it is necessary to carry out an effective anti-infection treatment. In this work, we designed and synthesized red blood cell (RBC) membrane-coated PLGA nanoparticles named γ3-RBCNPs, which target the highly expressed intercellular adhesion molecule-1 (ICAM-1) at the site of infection through the γ3 peptide on its surface and kill the *Klebsiella pneumoniae* through ciprofloxacin encapsulated in its core. In addition, the homogenous RBC membrane coated on the surface of the nanoparticles helps them avoid immune surveillance and prolong the circulation time of the drug in the body. We found that the γ3-RBCNPs target human umbilical vein endothelial cells (HUVECs) activated by TNF-α *in vitro* and the infected lung of mice in the sepsis model very well. *In vitro* evaluation suggested that γ3-RBCNPs have a low risk of acute hemolysis and are less likely to be engulfed by macrophages. *In vivo* evaluation showed that γ3-RBCNPs has a long half-life and good bio-safety. More importantly, we confirmed that γ3-RBCNPs have the good antibacterial and anti-infection ability *in vivo* and *in vitro*. Our research provides a new strategy for the nano-drug treatment of Klebsiella pneumoniae-induced sepsis.

## Introduction

Sepsis is caused by the invasion of pathogenic microorganisms into the blood circulation and causing systemic inflammatory response syndrome, which may further lead to acute respiratory distress, multiple organ dysfunction, circulatory failure, and even death (Napolitano, 2018). The incidence of sepsis is on the rise with the aging of the population, the increased incidence of tumors, and the increased use of invasive medical procedures. The global incidence of sepsis is increasing at a rate of 1.5–8.0% each year, of which more than 1/4 of patients died (Prescott and Angus, 2018). The pathogenesis of sepsis mainly lies in the massive release of inflammatory mediators such as tumor necrosis factor (TNF-α), interleukin (IL), platelet-activating factor (PAF), etc., caused by infection (Chousterman et al., 2017). These mediators further activate various cells and multiple organ systems throughout the body to produce a chain reaction and eventually lead to the loss of control of its defense mechanism (Huang et al., 2019).

All types of pathogenic microorganisms infection may cause sepsis, including Gram-negative bacteria, Gram-positive bacteria and fungi (Salomão et al., 2019). Among bloodstream infections caused by gram-negative bacteria, *Klebsiella pneumoniae* (*K.p.*) is the second most common cause after *Escherichia coli*, and the mortality is about 20–30% (Harris et al., 2018). The bloodstream infection caused by *K.p.* is a systemic infection that causes symptoms such as disseminated intra-vascular coagulation, multiple organ failure, and shock if the patients develop a severe infection (Bengoechea and Sa Pessoa, 2019). Many drugs have antimicrobial activity against *K.p.*, including the first to fourth generation cephalosporins, broad-spectrum penicillins, aminoglycoside antibiotics, fluoroquinolones, carbapenems, and monocyclic β-lactams (Fritzenwanker et al., 2018). However, in recent years, due to the excessive use of various antibiotics, *K.p.* has developed antibiotically resistance, which increases the difficulty in treating sepsis caused by *K.p.* In addition, the use of antibiotics in large doses may lead to adverse toxicity and side effects (Lee et al., 2017). Therefore, it is urgent to develop drugs with high efficiency, low toxicity, and fewer adverse reactions.

The key to combating antibiotic resistance is to improve the bioavailability of drugs in the infected lesions. Therefore, an effective delivery system is needed to enable high dose and sustained drug distribution in focal infection. In order to solve this problem, the nano-drug delivery system comes into being to achieve targeted delivery and sustained release of drugs. It has been proved that nano-drug delivery system can effectively prolong the blood circulation time of drugs, overcome the problem of insufficient actual drug dosage at the infection site, and minimize adverse side effects caused by the use of antibiotics (Gao et al., 2018; Ho et al., 2019). Besides, the bionic modification of the surface of the nano-drug delivery system endows it with different functions. For example, nanoparticles (NPs) functionalized with the red blood cell (RBC) membrane can avoid the recognition and phagocytosis of the immune system, prolong the circulation time of nanoparticles *in vivo*, and adsorb bacterial toxins and so on (Lee et al., 2019). In addition, platelet membranes and leukocyte membranes, can also be used to modify NPs functionally (Chen et al., 2019; Wang et al., 2020). Moreover, targeted release of drugs at the infection site can be achieved by imparting targeting properties to the nano-drug delivery system to improve the therapeutic efficiency further (Puttappa et al., 2019). There are two ways of targeted drug delivery based on NPs, passive and active targeting. The accumulation of bacteria in the infectious site stimulates the increase of various mediators involved in the inflammatory response, which will increase the permeability of blood vessels and promote the passive targeting of NPs (Ahmed et al., 2020). Active targeting can be achieved by modifying targeted antibodies, polypeptides, lectins, aptamers, and other molecules with targeted properties on the surface of NPs (Cui et al., 2019). In short, through reasonable modification of the nano-drug delivery system, the drug resistance can be resolved to a large extent, thereby achieving a better effect of treating sepsis.

In this article, we prepared γ3-RBCNPs for the treatment of sepsis caused by *K.p*. The γ3 peptide on the γ3-RBCNPs is a crucial element in the natural ligand of ICAM-1, which efficiently binds to ICAM-1 that is highly expressed in inflammatory tissues to achieve targeted drug delivery. The γ3-RBCNPs showed good targeting properties and therapeutic effects on infections caused by *K.p.*, providing a powerful method for treating sepsis. This work also provides a new idea for developing a nano-drug delivery system against other drug-resistant bacteria.

## Materials and Methods

### Materials

PLGA (Lactic acid: glycolic acid = 50:50, Mw = 1–100 KDa) was obtained from Beijing Top Science Biotechnology Co., Ltd. Poly(vinyl alcohol) (PVA), ciprofloxacin (CIP), dichloromethane, ethanol, paraformaldehyde, Triton-X100, formaldehyde were obtained from Aladdin Reagent (Shanghai) Co., Ltd. Phorbol 12-myristate 13-acetate (PMA), phosphotungstic acid, paraffin, fluorescent dyes Rhodamine 123 (Rho123), and cyanine3 (Cy3) were purchased from J&K Scientific (China). RPMI-1640 medium, Endothelial cell growth supplement (ECGS), RIPA lysis buffer, BCA protein assay kit, Tumor necrosis factor-α (TNF-α), and Bovine serum albumin (BSA) were purchased from Sigma Aldrich (United States). Fetal Bovine Serum (FBS) was purchased from Gibco (Thermo Fisher Scientific, United States). The DSPE-PEG-γ3 peptide was synthesized by Hangzhou Xinqiao Biotechnology Co., Ltd. Phosphate buffer saline (PBS) buffer, Tween-20, SDS-PAGE Preparation kit, Coomassie brilliant blue, LB Broth Powder, Protein marker, Annexin V Apoptosis Detection Kit (FITC/PI double staining), Hematoxylin-Eosin (HE) staining kit, Penicillin/Streptomycin (P/S) Solution, and Gentamycin sulfate solution were obtained from Shanghai Sangon Biotech (China). Endothelial Cell Medium (ECM) was purchased from Solarbio Life Sciences (Beijing, China). Hoechst33342 Staining Solution was obtained from Leagene Biotechnology (Beijing, China). Mouse TNF-α, IL-1β, and IL-6 ELISA kits were purchased from Beyotime Biotechnology (Shanghai, China). Caspase-1 Rabbit monoclonal antibody (ab207802) and Goat Anti-Rabbit IgG (Alexa Fluor^®^ 488) (ab150077) were purchased from Abcam (Cambridge, United Kingdom). The HUVEC cell line and THP-1 cell line were purchased from Tongpai Biotechnology (China). *Klebsiella pneumoniae* standard strain was purchased from National Center for Medical Culture Collections (CMCC).

### Preparation of PLGA@CIP NP-Cores

PLGA@CIP NP-cores were prepared by the water/oil/water (W/O/W) emulsion solvent evaporation method. First, 25 mg CIP was dissolved in 1 mL ddH_2_O to form the inner aqueous phase (W1), then 100 mg PLGA was weighed and dissolved in 4 mL dichloromethane to form the oil phase (O). Next, the W1 phase was added to the O phase, and the ultrasonic cell pulverizer (Misonix, United States) was applied for ultrasonic emulsification to form W1/O, then W1/O was immediately dropped to 60 mL outer aqueous phase (W2) that containing 2% PVA and magnetically stirred at 700 rpm for 4 h. Next, the prepared NPs solution was washed three times with ethanol and ddH_2_O successively and centrifuged at 3,000 rpm to collect the NPs. Finally, the precipitate was vacuum freeze-dried for 3 days to obtain PLGA@CIP NP-cores.

### Extraction and Modification of Red Blood Cell Membrane

Whole blood was collected from the mouse orbital venous plexus to prepare the RBC membrane. The collected whole blood was centrifuged (3,000 rpm, 5 min) to discard the upper layer, including plasma, leukocyte, and platelets. The obtained RBCs were washed with PBS 3 times to obtain packed RBCs. The packed RBCs were incubated in hypotonic 0.1 × PBS at 4°C for 30 min and centrifuged at 6,000 rpm for 5 min to discard the supernatant. Then the precipitate was washed repeatedly with 0.1 × PBS solution until the supernatant was colorless, and the RBC membrane (RBCm) was obtained.

Then the synthesized DSPE-PEG-γ3 peptide was added to the RBCm prepared above and incubated at a 37°C shaker for 1 h. Then the free DSPE-PEG-γ3 peptide was removed through centrifugation (6,000 rpm, 5 min), and the γ3-RBCm was obtained.

### Preparation of γ3-RBCNPs

γ3-RBCNPs were prepared by the membrane extrusion method. The obtained γ3-RBCm was mixed with PLGA@CIP NP-cores under ultrasound, and then the mixture was passed through a 200 nm polycarbonate porous membrane 20 times with the Avanti Mini-Extruder (United States). The obtained γ3-RBCNPs solution was centrifuged at 6,000 rpm for 10 min, washed with ethanol and ddH_2_O several times. The concentrated γ3-RBCNPs solution was stored at 4°C.

The synthesis method of fluorescently labeled γ3-RBCNPs@Rho123 and γ3-RBCNPs@Cy3 for *in vitro* and *in vivo* tracing was similar to the preparation of PLGA@CIP NP-cores, 1 mg/mL Rho123/Cy3 was added to the PLGA solution in order to prepare the fluorescently labeled PLGA NPs, the other steps were unchanged.

### Characterization of γ3-RBCNPs

Three batches of prepared γ3-RBCNPs were taken and diluted with ddH_2_O. The average particle size, particle size distribution and surface potential of γ3-RBCNPs were measured by Malvern laser particle size analyzer (Mastersizer, United Kingdom). The morphology of γ3-RBCNPs was observed by transmission electron microscopy (TEM) (JEM-F200, Japan Electronics, Japan). Briefly, the γ3-RBCNPs were diluted and dropped onto a copper net with a supporting film, and a filter paper absorbed the excess liquid. The samples were then stained with 2% phosphotungstic acid and dried. Finally, the morphology of the NPs was observed under the TEM.

### Assessment of the *in vitro* Drug Release of γ3-RBCNPs

First, 0.5, 1.0, 2.0, 3.0, 4.0, 5.0, 6.0, 7.0, 8.0, 9.0, and 10.0 mL of 20 mg/L CIP solution was accurately draw, and diluted to 10 mL with 0.1 mol/L hydrochloric acid solution. The absorbance of different concentrations of CIP at OD_277__nm_ was measured to obtain the standard curve of CIP.

Next, RBCNPs and γ3-RBCNPs solutions (5 mL) were injected into a dialysis bag and immersed in the beaker of PBS and stirred on a magnetic stirrer (37°C, 100 r/min). At present, 2 mL of PBS solution was drawn, and the absorbance at OD_277__nm_ was detected. Finally, the absorbance value at each time point was substituted into the standard curve equation to calculate the cumulative release percentage of CIP.

### Characterization of Proteins of RBC Membrane on γ3-RBCNPs

Sodium dodecyl sulfate-Polyacrylamide gel electrophoresis (SDS-PAGE) was used to characterize the RBCm surface proteins of γ3-RBCNPs. PIRA lysis buffer was added into PLGA@CIP NP-cores, RBCm, RBCNPs, and γ3-RBCNPs solution. The samples were shaken at 4°C for 15 min and centrifuged. Then, the total protein content of the supernatant was measured with the kit. Next, 20 μg samples were loaded to the gel, and the voltage was set as 150 V. After electrophoresis, the gel was stained in Coomassie brilliant blue solution for 1 h and decolorization. Finally, the gel was observed by an electrophoresis imaging system.

### Cell Culture

HUVEC and THP-1 cells were cultured and passaged with ECM medium and RPMI-1640 medium, respectively, with 10% FBS and 1% P/S solution supplement. In addition, the culture of HUVEC cells requires additional 1% ECGS. Cells were cultured in an incubator at 37°C and 5% CO_2_ and could be used for the further experiment after the third generation.

### Assessment of the *in vitro* Targeting Capacity of γ3-RBCNPs

Rho123-labeled γ3-RBCNPs were used to evaluate the *in vitro* targeting capacity of RBCNPs. First, HUVEC was cultured to an appropriate density, and 20 μg/mL TNF-α was added to the medium to construct a cell inflammation model. Then the PLGA@Rho123 NP-cores/RBCNPs/γ3–RBCNPs were added and incubated for 16 h. After being rinsed with PBS, the fluorescence of HUVEC cells in each group was recorded with Laser Scanning Confocal Microscope (LSCM) (Nikon, Japan).

In addition, the fluorescence intensity of each group of cells was analyzed by flow cytometry. First, the HUVEC cells were stimulated with TNF-α, and the negative control groups were added the same amount of PBS. Then PLGA@Rho123 NP-cores/RBCNPs/γ3-RBCNPs were incubated with the cells. After that, the cells were digested with trypsin, and the fluorescence intensity of Rho123 was detected by flow cytometry (BD FACSCalibur, United States).

### The Therapeutic Effect of γ3-RBCNPs on Intracellular Infection

The HUVEC was seeded in a 24-well plate and cultured until the cell confluence into a monolayer 1 day before the experiment. The *K.p.* was inoculated into LB medium, cultured at 37°C for 24 h and diluted with PBS for later use. The bacterial concentration was estimated by OD_600__nm_. Then, HUVEC was infected with *K.p.* according to bacteria: cells = 100: 1. After co-incubating for 2 h at 37°C, ECM containing 100 μg/mL gentamicin was added and incubated at 37°C for 1 h to kill the extracellular bacteria. Afterward, the cells were washed 4 times with PBS and incubated with PLGA@CIP NP-cores/RBCNPs/γ3-RBCNPs for 12 h. Next, after rinsing with PBS, 100 μL 0.5% Triton X-100 was added to lyse the cells for 8 min, and then 100 μL PBS was added immediately. The samples were repeatedly pipetted and gradient diluted (10^–1^∼10^–4^) with PBS. Finally, the diluted lysate was spread on LB plate and cultured until conspicuous colonies were formed, the number of colonies was counted.

### Flow Cytometry Detection

The process of HUVEC infected by *K.p.* and co-incubation with γ3-RBCNPs was the same as the previous description. HUVEC was collected and washed twice with pre-cooled PBS, the concentration of cells was adjusted to the same (1 × 10^6^/mL) by 1 × Binding Buffer. Then the cell suspensions were transferred into the flow tube, added with Annexin V-FITC and PI, and then incubated in the dark for 30 min. In the end, the cells were resuspended with 300 μL PBS and detected immediately by flow cytometry.

### Immunofluorescence Staining

HUVEC were seeded on the cell slide, the process of HUVEC infected by *K.p.* and co-incubation with γ3-RBCNPs was the same as above. After being rinsed with PBST, cells were fixed with 4% paraformaldehyde, permeabilized with 0.2% Triton X-100, and blocked with 5% BSA, successively. Then, the primary antibody anti-caspase-1 diluted with 1% BSA was used to incubate cells overnight at 4°C. The next day, cells were washed with PBST, the secondary antibody goat anti-rabbit (Alexa Fluor 488) diluted with 1% BSA was used to incubate cells at 37°C for 30 min in the dark. After being rinsed with PBST, 15 μL Hoechst solution was dropped on the cell slides and stained for 30 min in the dark. The fluorescence of cells was observed with LSCM.

### Hemolysis Test

Fresh blood from mice was used to prepare 2% RBC suspension. 12 tubes were divided into 4 groups, and 1.5 mL of RBC suspension was added to each tube. Next, 0.5 mL PBS/Triton-X100/RBCNPs/γ3-RBCNPs was added by group. The mixture was incubated in a 37°C water bath for 2 h, then centrifuged at 1,000 rpm for 5 min. The supernatant was transferred into a 96-well plate, and the absorbance at OD_545__nm_ was detected by a microplate reader (Biotek, China).

### Macrophage Phagocytosis Test

The THP-1 cells were seeded and cultured on slides in a 24-well plate. When the cells were wholly attached, the THP-1 cells were induced to differentiate into macrophages with 0.1 μg/mL PMA for 72 h. Then the macrophages were incubated with PLGA@Rho123 NP-cores/RBCNPs/γ3-RBCNPs for 0.5, 1, 2, and 4 h, respectively. In order to achieve intracellular tracing, PLGA NP-cores, RBCNPs, and γ3-RBCNPs were all labeled with Rho123. After the incubation, cells were washed and fixed, and then the cell slides were stained with Hoechst for microscopic imaging.

### Establishment of the Animal Model of Acute Sepsis Induced by *Klebsiella pneumoniae*

A total of 24 female Balb/c mice, weighing (25 ± 1) g, were purchased from Shanghai Slack Laboratory Animal Co., Ltd. All mice were raised in SPF environment, room temperature 20–22°C, humidity 40–70%, the light is alternated between light and shade for 12 h. All animal experiments are approved by Wuxi Fifth People’s Hospital. After anesthesia, the trachea of mouse was surgically exposed, and 0.04 mL bacterial solution with a concentration of 5 × 10^8^ CFU/mL was injected along the trachea quickly. Then, the mouse was slightly vertically shakened for 30 s to spread the bacterial solution evenly. After that, the skin of the mice was sutured, and the wound was disinfected.

### Evaluation of the *in vivo* Safety of γ3-RBCNPs

Thirty model mice were randomly divided into three groups and injected intravenously with PBS, PLGA@CIP NP-cores, and γ3-RBCNPs, respectively. Venous blood was drawn from the mice 24 h after injection, and the liver function and blood routine indexes of mice in each group were detected by an automatic biochemical analyzer (SHINOLA, China). In addition, the bodyweight of mice in each group was measured every day for seven consecutive days. On the 7th day, the mice were sacrificed, and the heart, liver, spleen, lung, and kidney were taken out, fixed with formalin, embedded with paraffin, and sliced for HE staining.

### Distribution of γ3-RBCNPs *in vivo*

Thirty model mice were divided into two groups, and 300 μL of RBCNPs-Cy3 and γ3-RBCNPs-Cy3 for *in vivo* tracking were injected intravenously. After 4, 16, and 24 h, five mice in each group were sacrificed, and the major organs were taken out. *In vivo* animal imaging system (Bruker, Germany) was used for imaging, and the fluorescence intensity in the major organs was quantified by image acquisition software (Molecular Image, MI).

### Half-Life of Drug in γ3-RBCNPs *in vivo*

The fluorescent dye Cy3 was used as a mimic drug. Cy3 standard was gradient diluted, the fluorescence intensity of different concentrations of Cy3 at 570 nm was detected, and the standard curve was drawn. Then, 12 model mice were divided into four groups and injected with Cy3, PLGA-Cy3 NP-cores, RBCNPs-Cy3, and γ3-RBCNPs-Cy3. After 10 and 30 min, 1, 3, 6, 12, and 24 h, venous blood of mice was drawn and centrifuged to detect the fluorescence intensity at 570 nm. The concentration of Cy3 in the blood was calculated according to the standard curve, and the half-life curve was drawn.

### Evaluation of the Therapeutic Effect of γ3-RBCNPs *in vivo*

Twenty model mice were divided into four groups and injected with PBS, PLGA@CIP NP-cores, RBCNPs, and γ3-RBCNPs, respectively. After 24 h, the mice were sacrificed, and venous blood was collected. Concentrations of the inflammatory factor TNF-α, IL-1β, and IL-6 in serum were determined by ELISA kit. In addition, HE staining was performed with the lung of mice to observe the degree of pulmonary infection. At the same time, the number of bacteria in the principal organs of the mice was quantified. Briefly, the mouse’s lung, liver, spleen, kidney, and blood were taken out aseptically, and the tissues were made into homogenate. Then, the tissue homogenate and blood were diluted in the same proportion and spread on LB plate. The number of colonies was counted after overnight incubation.

### Statistical Analysis

All experiments were done in triplicates, and the data were analyzed by SPSS 24.0 statistical software. The results were expressed as mean ± standard deviation. One-way analysis of variance was used to compare the differences. *P*-value of less than 0.05 was considered statistically significant.

## Results

### Characterization of γ3-RBCNPs

**FIGURE 1:**
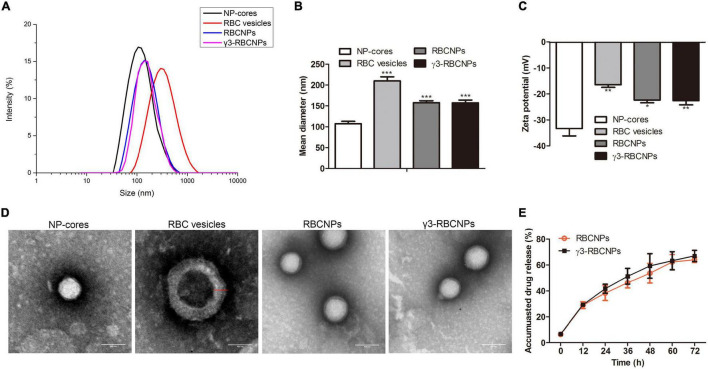
Characterization of γ3-RBCNPs. **(A,B)** DLS analysis of the size intensity **(A)** and mean diameter **(B)** of the prepared NPs. **(C)** Zeta potential of the prepared NPs. **(D)** TEM images of the NPs. Scale bar = 100 nm. **(E)** *In vitro* drug release curves of RBCNPs and γ3-RBCNPs. *P < 0.05, **P < 0.01, ***P < 0.001 vs. NP cores group.

We characterized the four prepared NPs, including PLGA@CIP NP-cores (NP-cores), RBC vesicles, RBCNPs and γ3-RBCNPs. The Malvern laser particle size analyzer was applied to measure the average size, size distribution, and surface potential of the NPs. The average diameter of the NP-cores was 108 nm, and the final functionalized γ3-RBCNPs was 157 nm (Figures 1A,B). Figure 1C showed the zeta potential, four kinds of NPs had a negative charge on their surface, and the zeta potential of γ3-RBCNPs was −22 mV. Figure 1D was the TEM images of the NPs. It could be seen that there was a membrane structure with a thickness of 50 nm on the surface of RBCNPs and γ3-RBCNPs, which was the same as that of RBC vesicles. In addition, we compared the *in vitro* drug release behavior of RBCNPs and γ3-RBCNPs by dialysis. As shown in Figure 1E, there was no significant difference in the drug release rate of RBCNPs and γ3-RBCNPs. Both RBCNPs and γ3-RBCNPs showed an excellent sustained release effect *in vitro*.

### Biological Characteristics of γ3-RBCNPs

**FIGURE 2:**
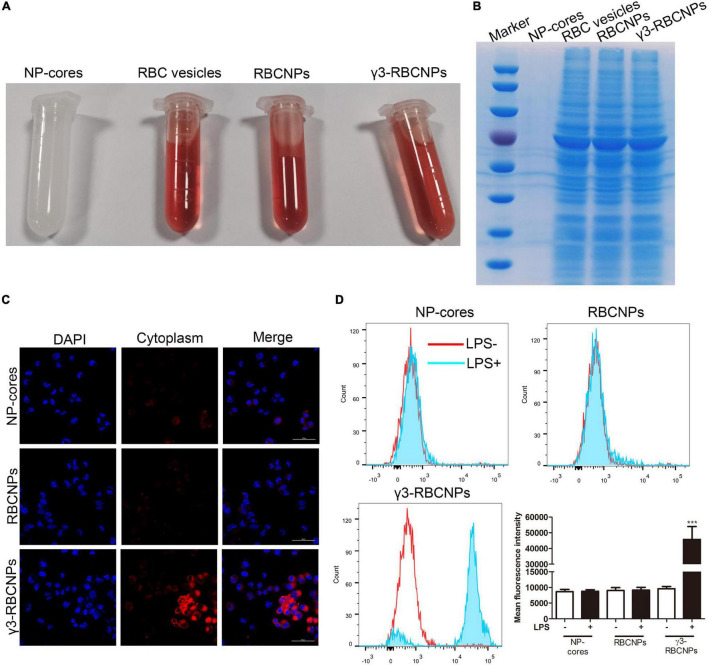
Biological characteristics of γ3-RBCNPs. **(A)** The appearance of the prepared NP-cores, RBC vesicles, RBCNPs, and γ3-RBCNPs. **(B)** SDS-PAGE analysis of the membrane protein of RBC on the surface of NP-cores, RBC vesicles, RBCNPs, and γ3-RBCNPs. **(C)** The targeting ability of NP-cores, RBCNPs, and γ3-RBCNPs on TNF-α-stimulated HUVECs was evaluated with LSCM. Scale bar = 50 μm. **(D)** The mean fluorescence intensity in HUVECs stimulated by LPS was assessed by flow cytometry. ***P < 0.001 vs. NP cores group.

From the appearance, NP cores are white emulsions, and the other three are all red because of the existence of RBCm (Figure 2A). Next, we identified the membrane proteins on the surface of these four NPs. As shown in Figure 2B, NP-cores did not contain any protein because its surface was not coated with RBCm, while the protein bands of RBCNPs and γ3-RBCNPs were consistent with RBC vesicles, indicating that the surface of RBCNPs and γ3-RBCNPs had been successfully coated with RBCm. Finally, in order to investigate whether RBCNPs coupled with γ3 peptides on the surface could target inflammatory cells *in vitro*, we used TNF-α to stimulate HUVEC cells to construct an inflammatory cell model, and Rho123 labeled NP-cores, RBCNPs and γ3-RBCNPs for intracellular tracing. The results are shown in Figure 2C. After treatment with NP-cores or RBCNPs, HUVEC cells showed less red fluorescence, while the large amount of HUVEC cells treated with γ3-RBCNPs showed red fluorescence.

In addition, we used flow cytometry to quantify the fluorescence intensity of Rho123 in each group of HUVEC cells. For the NP-cores and RBCNPs groups, LPS stimulation had no significant effect on the change of intracellular fluorescence intensity. While for the γ3-RBCNPs group, the fluorescence intensity in the HUVEC cells stimulated by LPS was significantly higher than that of unstimulated cells (Figure 2D). The above results indicated that γ3-RBCNPs are capable of targeting TNF-induced HUVECs.

### *In vitro* Therapeutic Effects of γ3-RBCNPs

**FIGURE 3:**
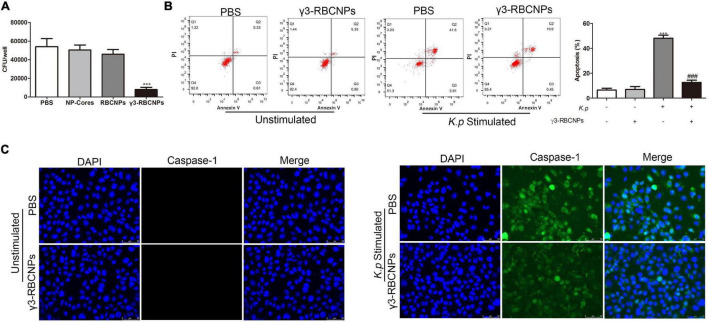
Evaluation of the therapeutic effect of γ3-RBCNPs *in vitro*. **(A)** Quantification of the number of bacteria in HUVECs treated with NP-cores, RBCNPs, and γ3-RBCNPs after *K.P* infection. ***P < 0.001 vs. PBS group. Flow cytometry **(B)** and IF staining **(C)** detect the apoptosis of HUVECs treated with γ3-RBCNP after *K.P* infection. Scale bar = 50 μm. ***P < 0.001 vs. unstimulated groups. ^###^*P* < 0.001 vs. *K.P* stimulated PBS group.

In order to evaluate the *in vitro* therapeutic effect of γ3-RBCNPs on *K.P* infection, we co-cultured HUVEC cells with *K.P* and treated them with PBS/NP-cores/RBCNPs/γ3-RBCNPs. The number of bacteria in the cells was quantified. After being treated with γ3-RBCNPs, the number of bacteria decreased significantly compared with the PBS group, while the therapeutic effects of NP-cores and RBCNPs were not obvious (Figure 3A).

We also conducted flow cytometry to detect cell apoptosis. When there was no *K.P* infection, the percentage of HUVEC apoptosis was small with or without the addition of γ3-RBCNPs, indicating that γ3-RBCNPs were less cytotoxic. When only infected with *K.P*, the apoptotic rate of HUVEC was about 50%, but after being treated with γ3-RBCNPs, the apoptotic rate decreased significantly (Figure 3B).

Moreover, we performed IF staining of Caspase-1 in the infected HUVEC cells (Figure 3C). Compared with the uninfected group, the Caspase-1 activity was high in the cells infected with *K.P* and treated with PBS. At the same time, it was significantly decreased (43.58% intensity decrease) in the cells treated with γ3-RBCNPs after *K.P* infection.

### *In vivo* Targeting Capacity of γ3-RBCNPs

**FIGURE 4:**
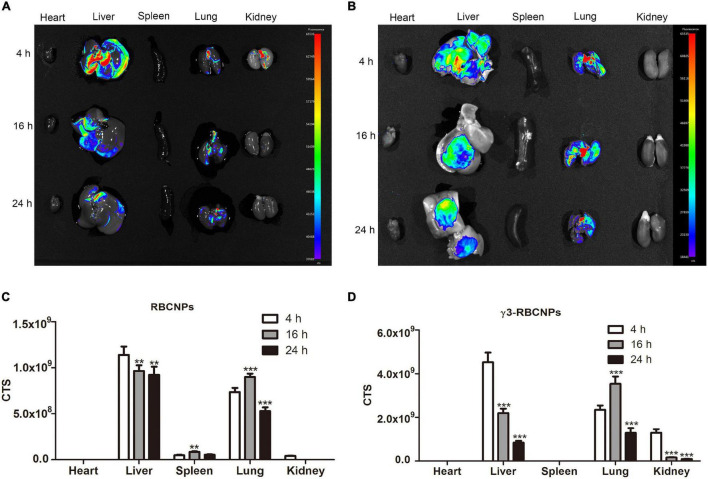
The targeting capacity of γ3-RBCNPs *in vivo*. Drug distribution in major organs of mice with acute pulmonary infection treated with RBCNPs **(A)** and γ3-RBCNPs **(B)** at 4, 16, and 24 h. Quantification of the fluorescence intensity in the main organs of mice in RBCNPs **(C)** and γ3-RBCNPs **(D)** groups. **P < 0.01 vs. 4 h group. ***P < 0.001 vs. 4 h group.

We have previously demonstrated that γ3-RBCNPs strongly target TNF-α-stimulated HUVEC. To investigate whether γ3-RBCNPs also target TNF-α-stimulated HUVEC *in vivo*, we established a mouse model of acute pulmonary infection induced by *K.P*. To facilitate *in vivo* tracing, RBCNPs and γ3-RBCNPs were labeled with the near-infrared fluorescent dye Cy3 and intravenously injected into the mice, followed by fluorescence imaging of the major organs of the mice at different time points (Figures 4A,B). Fluorescence intensity in these major organs of the two groups of mice was also quantified (Figures 4C,D). At the timing of 4, 16, and 24 h, the fluorescence intensity of the lung of mice injected with γ3-RBCNPs was significantly higher than that injected with RBCNPs, indicating that γ3-RBCNPs had the best targeting capacity *in vivo*.

### *In vitro* Evaluation of γ3-RBCNPs

**FIGURE 5:**
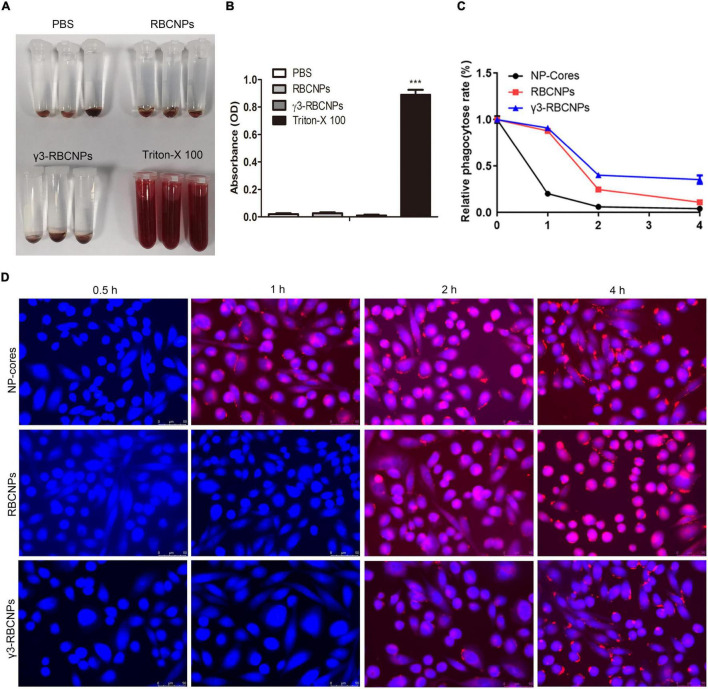
*In vitro* evaluation of γ3-RBCNPs. **(A,B)** Assessment of the hemolytic activity of NP-cores, RBCNPs, and γ3-RBCNPs, PBS was used as the negative control, and Triton-X100 was used as a positive control. **(C,D)** The phagocytosis of RBCNPs and γ3-RBCNPs by macrophages *in vitro* was observed by LSCM. Scale bar = 50 μm. ***P < 0.001 vs. PBS group.

γ3-RBCNPs were synthesized to treat sepsis by direct intravenous injection, and their safety must be considered, so we evaluated their potential hemolysis. There experiments were divided into four groups, PBS, Triton-X100, RBCNPs, and γ3-RBCNPs groups. PBS group was the negative control (NC), and the Triton-X100 group was a positive control (PC). The results are shown in Figures 5A,B.

In order to verify whether it could avoid the phagocytosis of macrophages, we used PMA to induce THP-1 cells to differentiate into macrophages and then observed the phagocytosis of γ3-RBCNPs. As shown in Figures 5C,D, NP-cores were 79.59% phagocytosed by macrophages at 1 h, 75.14% of RBCNPs were phagocytosed at 2 h, and only 59.79% of γ3-RBCNPs was phagocytosed at 2 h. This result indicates that modification of NPs with RBCm effectively helps NPs avoid phagocytosis by macrophages. Thus, enhancing the therapeutic effect.

### Evaluation of the Bio-Safety of γ3-RBCNPs *in vivo*

**FIGURE 6:**
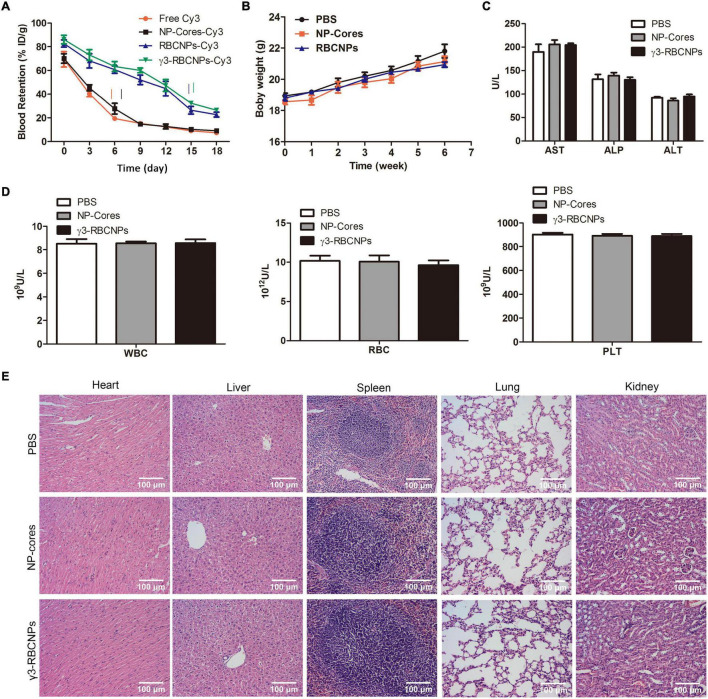
Assessment of the bio-safety of γ3-RBCNPs *in vivo*. **(A)** Evaluation of half-life of the drug in NP-cores, RBCNPs, and γ3-RBCNPs *in vivo*. The changes of body weight **(B)**, liver function **(C)** and blood routine **(D)** indexes of mice with acute pulmonary infection treated with NP-cores, RBCNPs, and γ3-RBCNPs. **(E)** H&E staining of the main tissues in mice. Scale bar = 100 μm.

Although γ3-RBCNPs have been confirmed to have targeted therapeutic efficacy *in vivo*, the bio-safety of γ3-RBCNPs still needs to be evaluated. To this end, we first detected the half-life of γ3-RBCNPs *in vivo*, Cy3-labeled NP-cores, RBCNPs, and γ3-RBCNPs were used for intravenous injection. Then, the half-life was calculated by detecting fluorescence values in blood at multiple time points. The results showed that free Cy3 and NP-cores-Cy3 quickly reached the half-life, while the half-life of RBCNPs-Cy3 and γ3-RBCNPs-Cy3 was significantly prolonged (Figure 6A). In addition, NP-cores, RBCNPs and γ3-RBCNPs were intravenously injected into the mice. The body weight of the mice in each group was measured daily, and it was the same during the treatment period (Figure 6B). At the same time, the liver function (AST, ALP, ALT) and blood routine indexes (WBC, RBC, PLT) were detected. There was no significant difference in the indexes of the mice in each group (Figures 6C,D). Finally, the HE staining of the major organs sections was performed, it can be seen that the major organs of the mice in each group were not damaged (Figure 6E).

### *In vivo* Therapeutic Effects of γ3-RBCNPs

**FIGURE 7:**
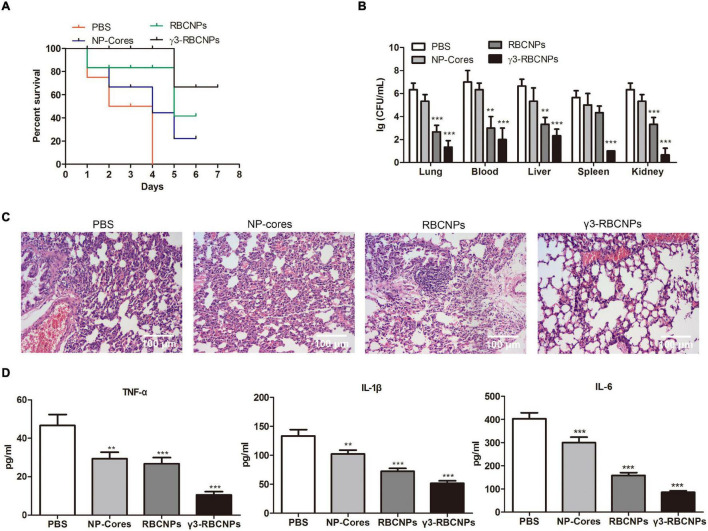
Evaluation of the therapeutic effect of γ3-RBCNPs *in vivo*. **(A)** Survival curves of mice with an acute lung infection in each group after being treated with NP-cores, RBCNPs, and γ3-RBCNPs. **(B)** Quantification of bacterial counts in main tissues of mice after treatment. **(C)** HE staining was performed to evaluate the degree of lung infection in mice after treatment. Scale bar = 100 μm. **(D)** ELISA assay was conducted to detect the level of TNF-α, IL-1β, and IL-6 in the serum of mice. **P < 0.01 vs. PBS group, ***P < 0.001 vs. PBS group.

The model mice were injected intravenously with PBS, NP-cores, RBCNPs, and γ3-RBCNPs, respectively, then the therapeutic effects were evaluated. From the survival curve of mice (Figure 7A), we found that γ3-RBCNPs significantly prolong the survival of mice with sepsis. Furthermore, compared with the PBS group and the NP-cores group, the number of bacteria in the main organs of the γ3-RBCNPs group was significantly reduced (Figure 7B), indicating that γ3-RBCNPs effectively inhibit the proliferation of *K.P*. In addition, HE staining of lung tissue showed that different degrees of inflammatory cell infiltration and tissue edema was presented in the PBS group, NP-Cores group and RBCNPs group. In contrast, the lung tissue infection was significantly improved in the γ3-RBCNPs group (Figure 7C). Besides, the serum levels of TNF-α, IL-1β, and IL-6 in the γ3-RBCNPs group were significantly lower than those in PBS groups (Figure 7D), indicating that γ3-RBCNPs reduced systemic inflammatory response in mice with sepsis.

## Discussion

In recent years, *K.p.* has become an important pathogen of nosocomial infection. Sepsis caused by *K.p.* is a systemic infection that leads to severe symptoms such as disseminated intravascular coagulation failure and shock (Wyres and Holt, 2018). At present, the emergence of drug-resistant *K.p.* strains leads to the gradual deterioration of the therapeutic effect of antibiotics (Vuotto et al., 2017). At the same time, large doses of antibiotics also have greater side effects and toxicity. The application of a nano-drug delivery system may be a good solution to these problems. Hussain et al. (2018) developed a targeted nano-silicon drug delivery system loaded vancomycin through its porous silicon spheres, effectively inhibiting Staphylococcus aureus infection. In this study, we used biodegradable PLGA to deliver the quinolone antibiotic CIP directly to the site of infection, which showed a good sustained drug release effect and prolonged half-life (Figures 1E, 6A).

Recently, multiple studies have been conducted on the targeted treatment of sepsis with nano-technology (Lou et al., 2018; Yuk et al., 2018; Choi et al., 2020). For example, Zhang et al. (2018) developed a dual-response NPs that targeted the infection site to treat sepsis. The core of the NPs could respond to pH/bacterial enzymes of the infected microenvironment and release the drug. In addition, the ICAM-1 antibody on the surface of the core could directly target the infected tissue. Yang et al. (2020) also developed a nano-drug delivery system that targets ICAM-1 at the infected tissue under the γ3 peptide. Although these reports provide new ideas for applying nano-technology in the targeted treatment of sepsis, there are still some drawbacks. For example, the stability of these nano-drug delivery systems in the body is insufficient because they are easily recognized by the immune system as invaders and then removed, thus reducing the treatment efficiency. To solve this problem, in this study, we made some improvements based on the existing researches. Through biological modification, we coated the surface of PLGA@CIP with a homologous RBC membrane to improve the stability *in vivo*. The solution of RBCm coated NPs was all red, and the NPs were verified to avoid phagocytosis by macrophage (Figure 5C). This means that the NPs possess the invisibility capacity during circulation in the blood, thus escaping the immune surveillance.

The synthesized γ3-RBCNPs achieve targeted therapy using the specific binding ability of γ3 peptide to ICAM-1 at the infection site. The excellent targeting ability was validated in TNF-α-stimulated HUVEC cells and acute lung *K.p*.-infected mice. On the one hand, this targeting function is conducive to the accumulation of drugs at the infection site, and on the other hand, it can also minimize the systemic toxicity of drugs. Furthermore, we verified that γ3-RBCNPs did have good bio-safety and did not cause hemolysis and damage to organs such as the liver and kidney in mice. This is essential for the clinical application of nanomedicine. In addition, γ3-RBCNPs could reduce the number of bacteria in *K.p.*-infected cells and tissues, inhibit the apoptosis of infected cells, and reduce the systemic inflammatory response in mice with acute sepsis, indicating the γ3-RBCNPs has a very good therapeutic effect and is worth spreading. However, there are certain limitations in our research. We used *K.p.* to induce acute lung infection in mice as an animal model of sepsis. We confirmed that γ3-RBCNPs have the good antibacterial and anti-infection ability *in vivo* and *in vitro*. Our research provides a new strategy for the nano-drug treatment of Klebsiella pneumoniae-induced sepsis. However, it is still necessary to conduct systematic research on the choice of animal, the duplication methods and evaluation indicators when used in clinical research.

## Conclusion

In summary, the synthesized γ3-RBCNPs can efficiently load the fluoroquinolone drug ciprofloxacin, be enriched in the infected sites through passive and active targeting, and avoid the uptake of macrophages at the same time. *In vitro* experiments proved that γ3-RBCNPs significantly inhibit the proliferation of intracellular bacteria and promote the apoptosis of cells infected by *K.p.* In addition, for acute lung infections caused by *K.p.*, γ3-RBCNPs also showed perfect targeting and therapeutic effects. γ3-RBCNPs significantly prolong the half-life of the loaded drug ciprofloxacin, and with good biological safety. Our research provides a new strategy for the treatment of sepsis caused by *K.p.*

## Data Availability Statement

The datasets presented in this study can be found in online repositories. The names of the repository/repositories and accession number(s) can be found below: https://www.jianguoyun.com/p/Dbt-RVMQp4rPChiq38IEIAA; https://www.jianguoyun.com/p/DeOo4PAQp4rPChi238IEIAA; https://www.jianguoyun.com/p/DXeMfX0Qp4rPChi338IEIAA; https://www.jianguoyun.com/p/DTU7rG0Qp4rPChi438IEIAA; https://www.jianguoyun.com/p/DcxMiqwQp4rPChi938IEIAA; https://www.jianguoyun.com/p/DSHU_JoQp4rPChjA38IEIAA; and https://www.jianguoyun.com/p/DVQHQn8Qp4rPChjB38IEIAA.

## Ethics Statement

The animal study was reviewed and approved by the Animal Ethics Committee of Wuxi Fifth People’s Hospital.

## Author Contributions

YJ, XR, and SZ conceived and designed the study. JL, HD, and MZ performed the literature search and data extraction. FT, TH, and LZ drafted the manuscript. All authors read and approved the final manuscript.

## Conflict of Interest

The authors declare that the research was conducted in the absence of any commercial or financial relationships that could be construed as a potential conflict of interest.

## Publisher’s Note

All claims expressed in this article are solely those of the authors and do not necessarily represent those of their affiliated organizations, or those of the publisher, the editors and the reviewers. Any product that may be evaluated in this article, or claim that may be made by its manufacturer, is not guaranteed or endorsed by the publisher.
