# Supplementary material for: Functionalized Erythrocyte Membrane-Coated Nanoparticles for the Treatment of Klebsiella pneumoniae-Induced Sepsis
Source: Front Microbiol. 2022 Jun 16;13:901979. doi: 10.3389/fmicb.2022.901979 (PMC9244542; doi:10.3389/fmicb.2022.901979)
Supplement: Supplementary file 1 [file Data_Sheet_1.PDF]

Fig.1D

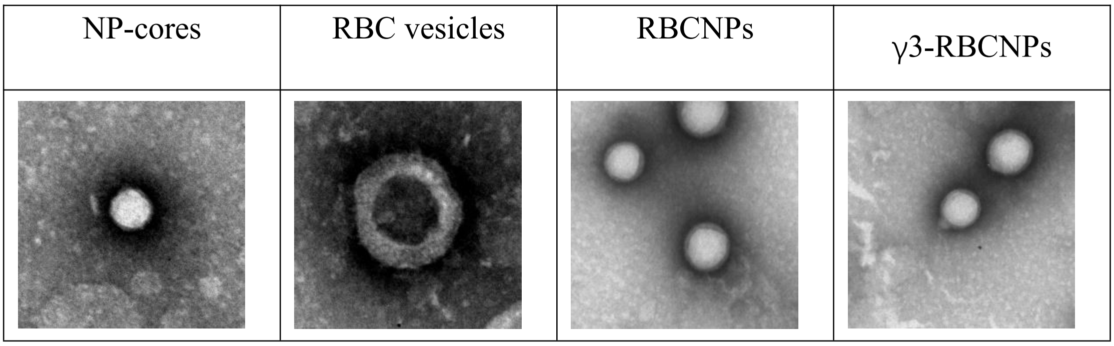

Fig.2A

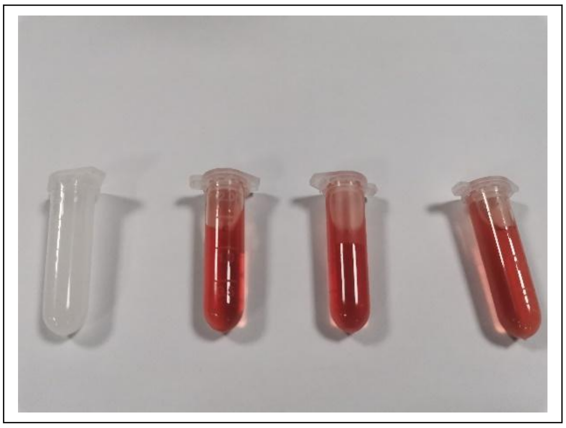

Fig.2B

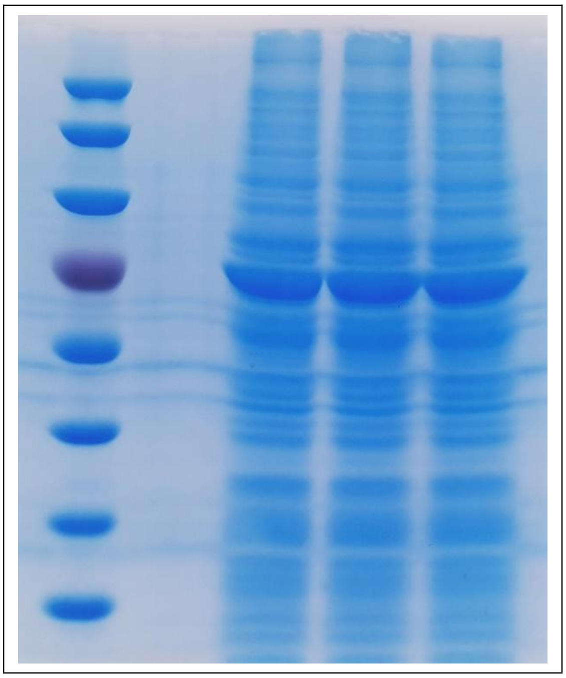

Fig.2C

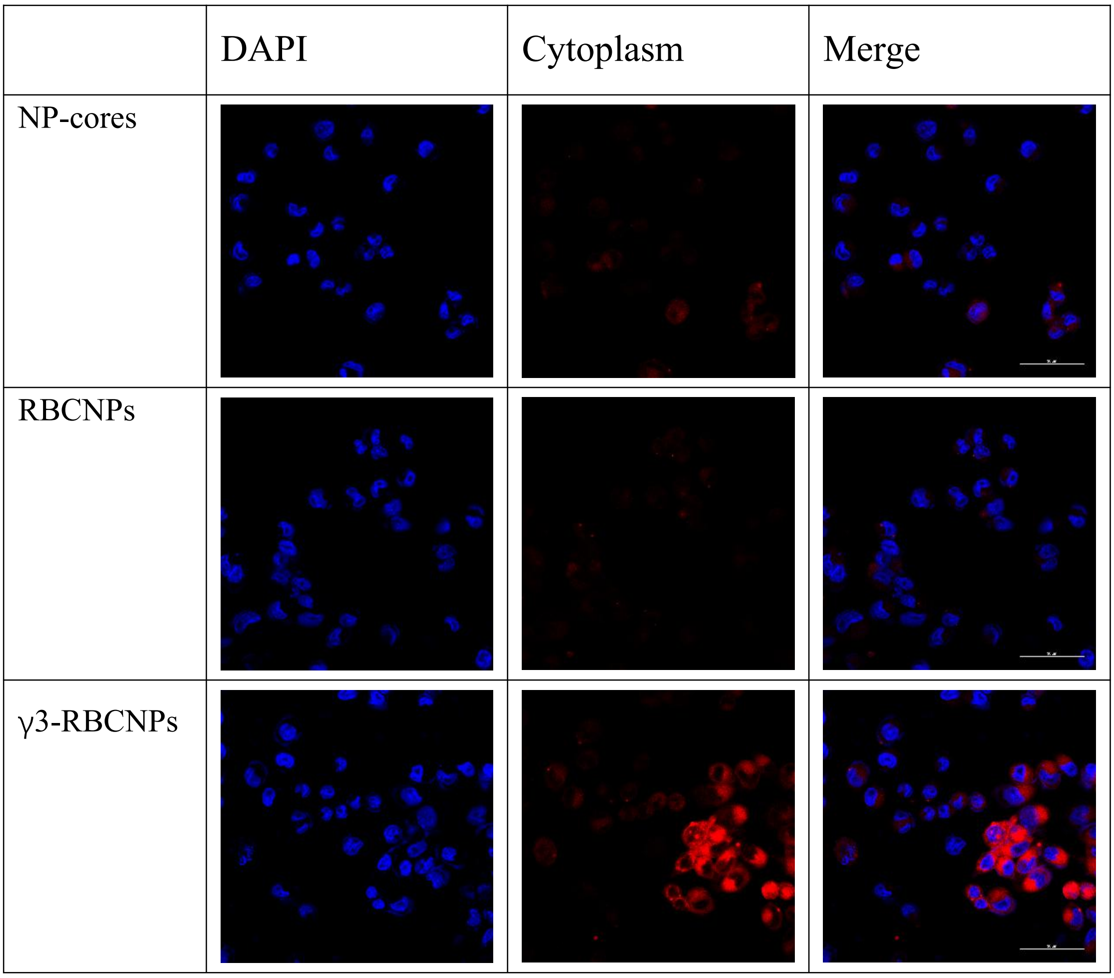
DAPI
Cytoplasm
Merge
NP-cores
RBCNPs
γ3-RBCNPs

Fig.2D

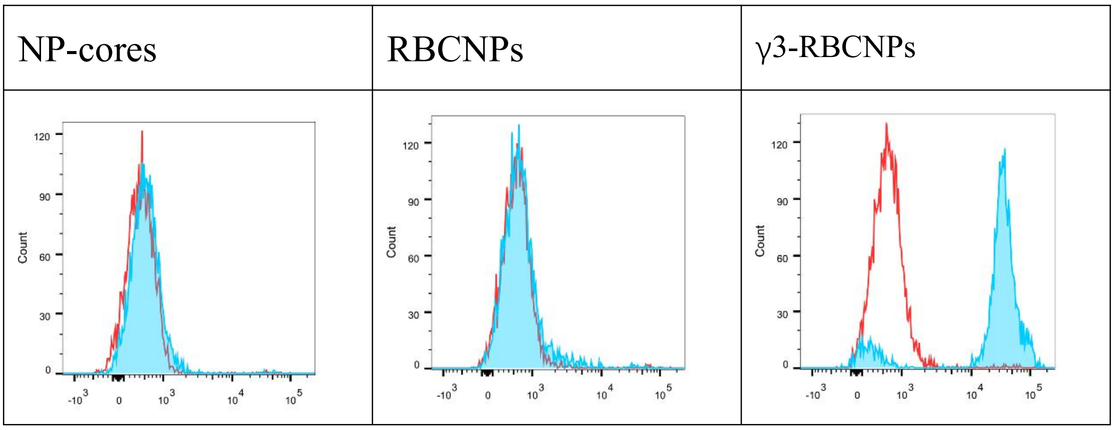
NP-cores
RBCNPs
γ3-RBCNPs
Count
120
90
60
30
0
-10^3
0
10^3
10^4
10^5

Fig.3B

<table>
<tr><td></td><td>PBS</td><td>γ3-RBCNPs</td></tr>
<tr><td>Unstimulated</td><td>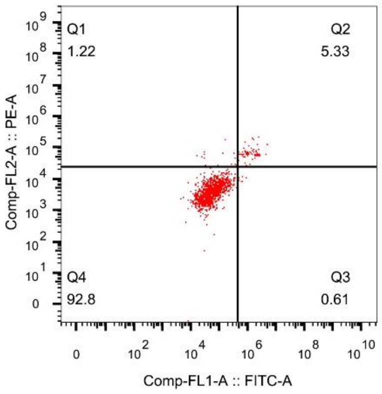
</td><td>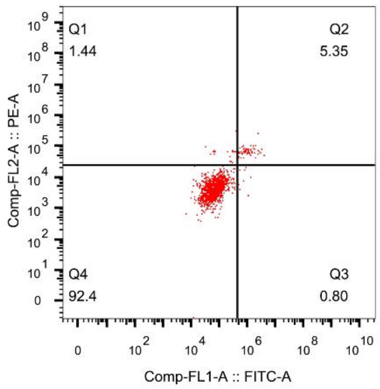
</td></tr>
<tr><td>k.p Stimulated</td><td>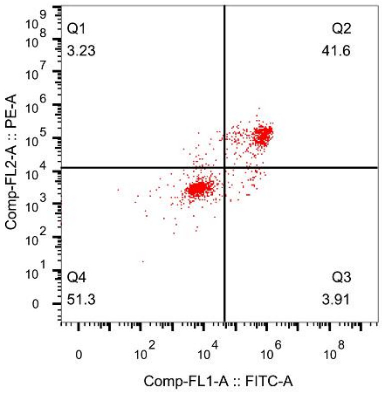
</td><td>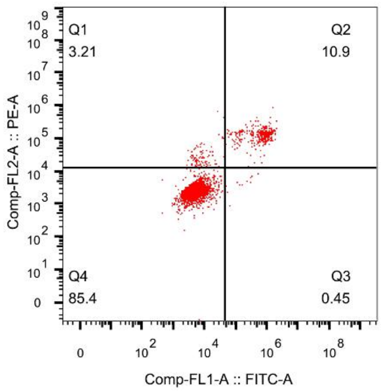
</td></tr>
</table>

Fig.3C

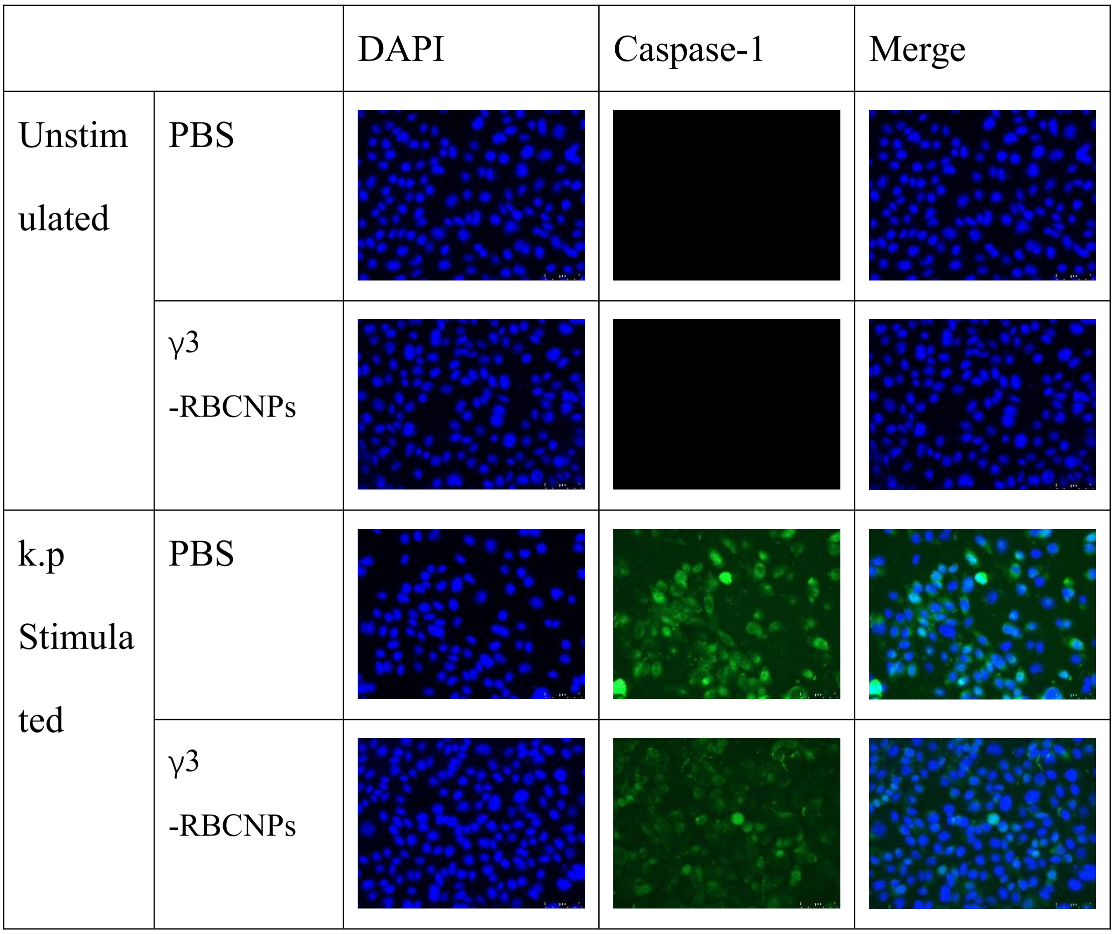
DAPI
Caspase-1
Merge
Unstimulated
PBS
γ3 -RBCNPs
k.p Stimulated
PBS
γ3 -RBCNPs

Fig.4A

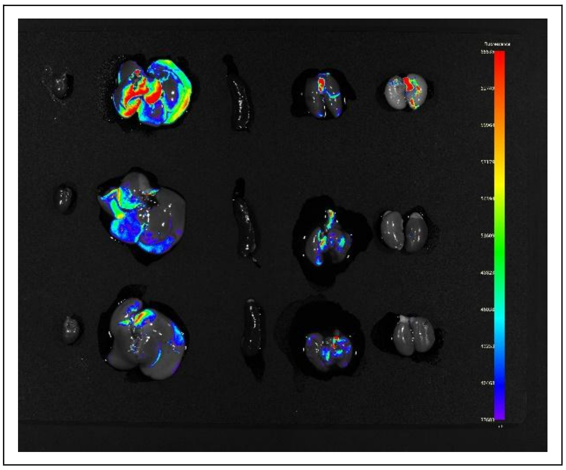

Fig.4B

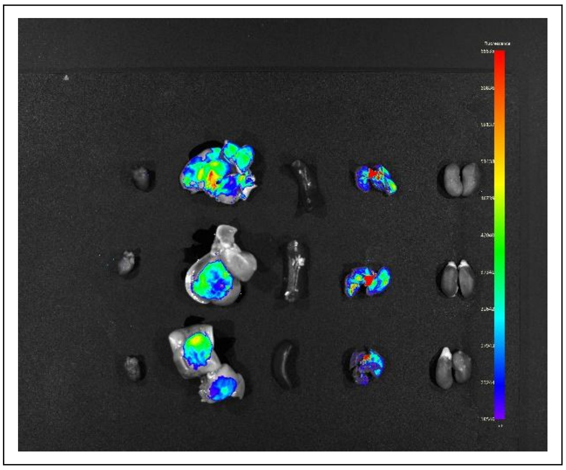

Fig.5A

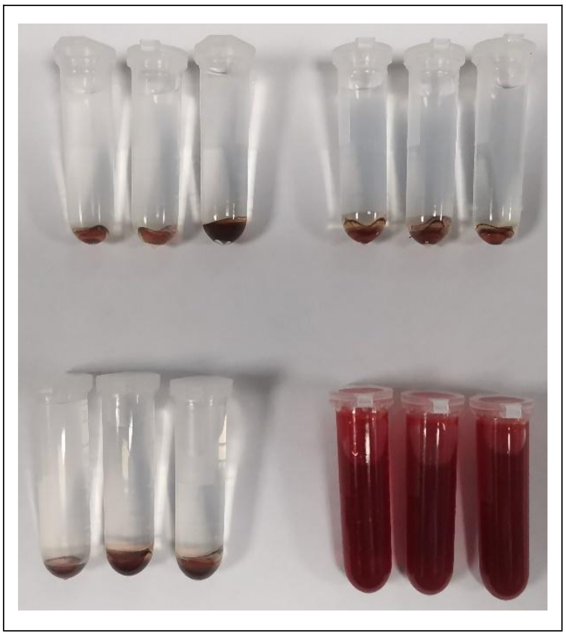

Fig.5C

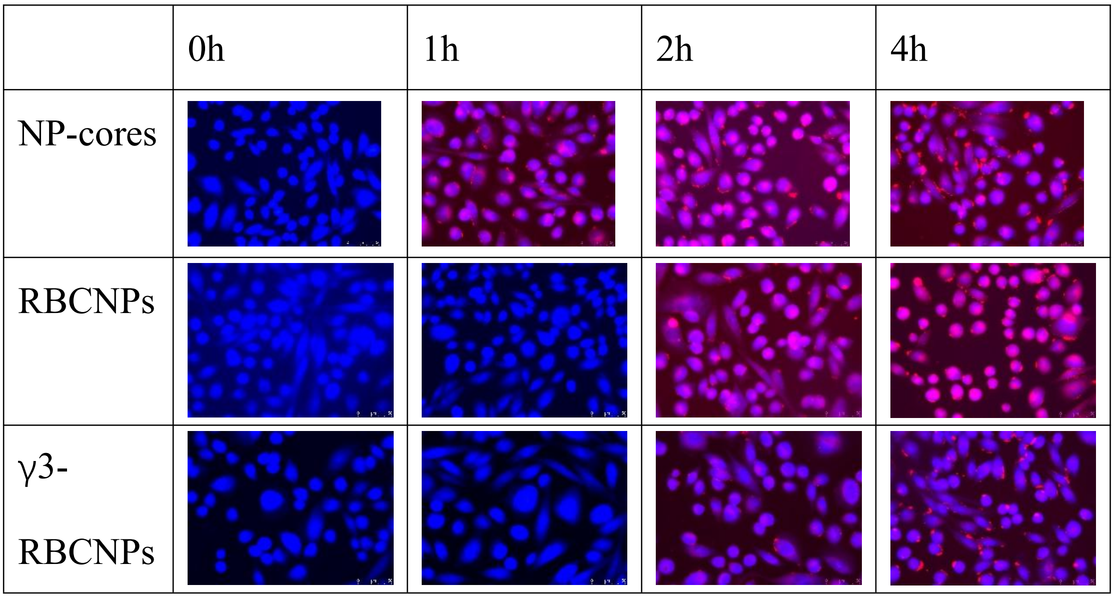

Fig.6E

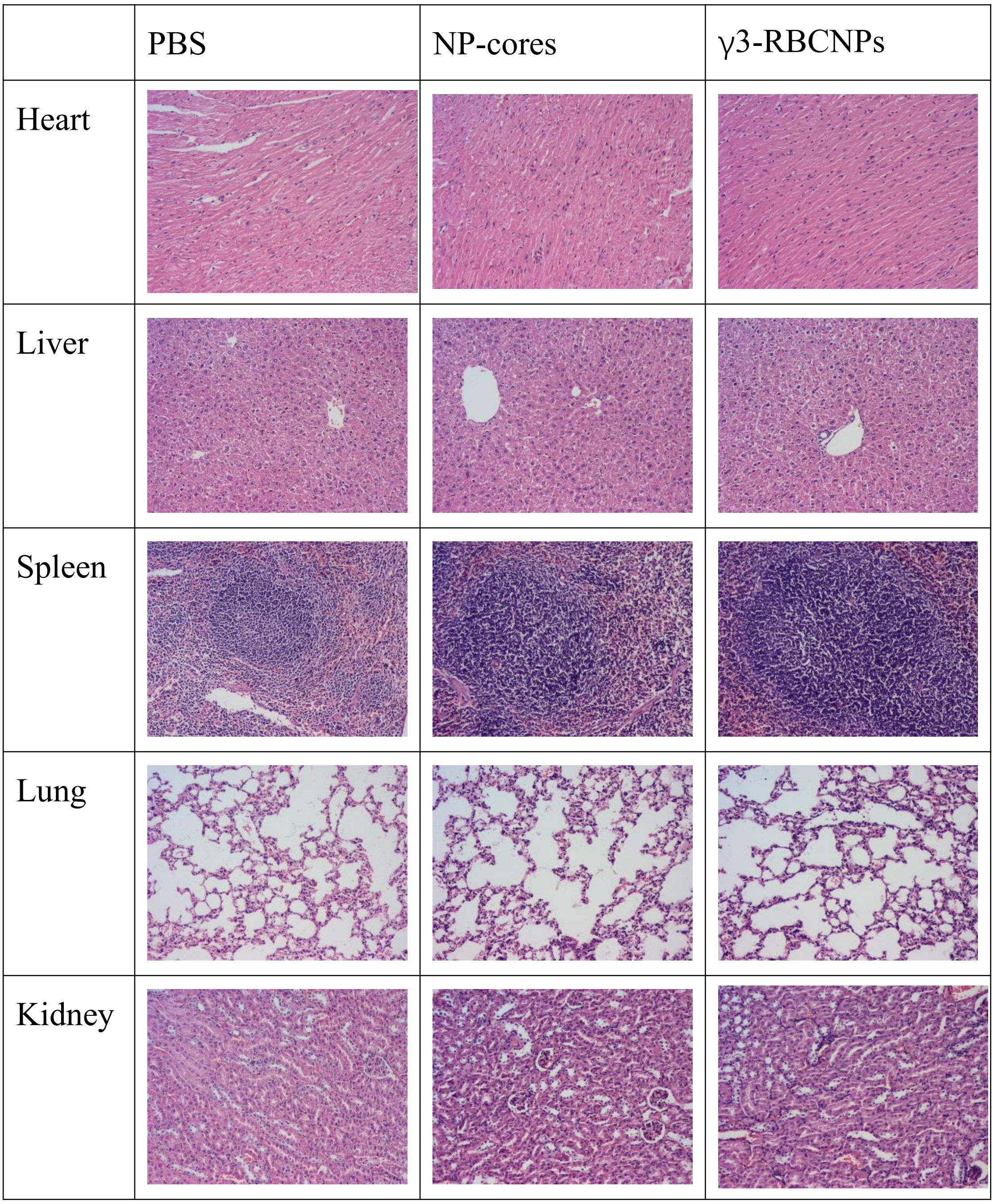

Fig.7C

PBS	NP-cores	RBCNPs	γ3-RBCNPs
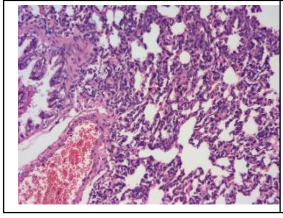	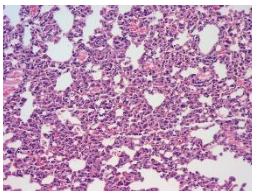	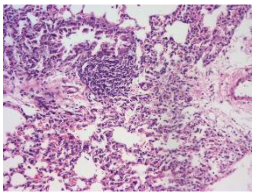	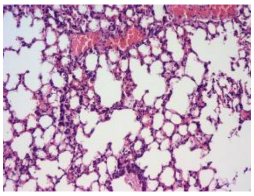
